# Acid Ceramidase Promotes Nuclear Export of PTEN through Sphingosine 1-Phosphate Mediated Akt Signaling

**DOI:** 10.1371/journal.pone.0076593

**Published:** 2013-10-01

**Authors:** Thomas H. Beckham, Joseph C. Cheng, Ping Lu, S. Tucker Marrison, James S. Norris, Xiang Liu

**Affiliations:** Department of Microbiology and Immunology, Medical University of South Carolina, Charleston, South Carolina, United States of America; University of California Irvine, United States of America

## Abstract

The tumor suppressor PTEN is now understood to regulate cellular processes at the cytoplasmic membrane, where it classically regulates PI3K signaling, as well as in the nucleus where multiple roles in controlling cell cycle and genome stability have been elucidated. Mechanisms that dictate nuclear import and, less extensively, nuclear export of PTEN have been described, however the relevance of these processes in disease states, particularly cancer, remain largely unknown. We investigated the impact of acid ceramidase on the nuclear-cytoplasmic trafficking of PTEN. Immunohistochemical analysis of a human prostate tissue microarray revealed that nuclear PTEN was lost in patients whose tumors had elevated acid ceramidase. We found that acid ceramidase promotes a reduction in nuclear PTEN that is dependent upon sphingosine 1-phosphate-mediated activation of Akt. We were further able to show that sphingosine 1-phosphate promotes formation of a complex between Crm1 and PTEN, and that leptomycin B prevents acid ceramidase and sphingosine 1-phosphate mediated loss of nuclear PTEN, suggesting an active exportin-mediated event. To investigate whether the tumor promoting aspects of acid ceramidase in prostate cancer depend upon its ability to export PTEN from the nucleus, we used enforced nuclear expression of PTEN to study docetaxel-induced apoptosis and cell killing, proliferation, and xenoengraftment. Interestingly, while acid ceramidase was able to protect cells expressing wild type PTEN from docetaxel, promote proliferation and xenoengraftment, acid ceramidase had no impact in cells expressing PTEN-NLS. These findings suggest that acid ceramidase, through sphingosine 1-phosphate, promotes nuclear export of PTEN as a means of promoting tumor formation, cell proliferation, and resistance to therapy.

## Introduction

PTEN is a critically important tumor suppressor classically known to antagonize oncogenic PI3K/Akt signaling by dephosphorylating the lipid product of PI3K, PIP_3,4,5_, thereby antagonizing pleckstrin homology domain dependent recruitment of Akt and its activating kinase PDK1 to the cell membrane [1,2]. This function, while undoubtedly a key factor in PTEN-mediated tumor suppression, is by no means the only described role for PTEN with interest in recent years focusing on the role of PTEN within the nucleus. Nuclear PTEN is now known to serve lipid-phosphatase-independent functions in regulating the cell cycle by promoting acetylation of p53 and upregulating RAD51 in response to DNA damage [3], and by mediating APC/C tumor suppression by promoting association with the adaptor CDH1 [4]. Besides these molecular functions, nuclear PTEN has been observationally linked to tumor suppression. Histological analysis of PTEN has shown that nuclear PTEN in tumor tissue was a favorable prognostic indicator and correlated with a lower tumor proliferation index in melanoma and colon cancer tissues [5,6]. Interestingly, the most frequent mutation in the hamartomarous condition Cowden Syndrome, in which patients inherit a mutant PTEN allele and are susceptible to cancer, is Lysine289. This mutant form retains its phosphatase activity, but is not imported into the nucleus, providing strong suggestive evidence that nuclear PTEN is important in suppression of neoplasia [7,8].

Several studies describe mechanisms that mediate import of PTEN into the nucleus including active import based on multiple cryptic nuclear localization- signal-like sequences, mutation of which abrogated RAN-mediated [9] or Major Vault Protein-mediated [10,11] nuclear accumulation of PTEN; PTEN C-terminus phosphorylation [12]; and monoubiquitination [7]. In contrast, little is known about active mechanisms of export of PTEN from the nucleus. One report by Liu, et al, showed that PTEN is exported from the nucleus at the G1/S transition through Akt-mediated activation of S6K [13]. They showed a direct interaction of PTEN with S6K and suggested this is mediated by the master nuclear export protein Crm1.

Here we report Crm1-dependent export of nuclear PTEN in response to sphingosine 1-phosphate (S1P) signaling. We found that expression of acid ceramidase (AC) in prostate cancer cells promoted a loss of nuclear PTEN. Following our recent study outlining AC-mediated Akt activation [14], we determined that AC-induced Akt activation promoted nuclear export of PTEN. Furthermore, we show that S1P strongly promotes formation of a complex between PTEN and Crm1, and that inhibition of Crm1 with Leptomycin B prevents AC/S1P-mediated export of nuclear PTEN. Interestingly, while AC was capable of promoting cell proliferation and resistance to Docetaxel in cells expressing wild type PTEN, it was not able to do so in cells expressing PTEN-NLS (wild type PTEN with an N-terminal nuclear localization signal attached), suggesting that the oncogenic properties of AC in prostate cancer involve its ability to regulate the level of PTEN in nucleus. Because most prostate cancers overexpress AC, we report a disease-relevant active mechanism of AC-mediated nuclear PTEN insufficiency promoting prostate cancer.

## Materials and Methods

### Cell lines and culture

PPC-1 [15] (a kind gift of Dr. Yi Lu, University of Tennessee), 22rv1, and DU145 (ATCC, Manassas, VA) were maintained in RPMI 1640 media supplemented with 10% bovine growth serum and incubated in 5% CO2 at 37°C. DU145-AC-EGFP/DU145-EGFP have been described [16,17].

### Reagents

Synthesis of S1P was conducted in the Lipidomics Shared Resource. S1P dry aliquots were prepared by suspension in methanol and lyophilization under a dry nitrogen stream. Aliquots were stored at -80°C until use at which time they were resuspended in PBS to 100µM. Reagents used include: SKI-II, Docetaxel (Thermo, Fisher, Lafayette, CO), LY294002, AktX (Cayman Chemical, Ann Arbor, MI), W146, JTE013 (Tocris, Bristol, UK), Leptomycin B (Sigma, St. Louis, MO), and S6K1 inhibitor DG2 (Millipore, Billerica, MA).

### Transfections and plasmid constructs

All plasmid transfections were carried out using Lipofectamine 2000 (Life Technologies) according to manufacturer instructions. The following plasmids were used in this study: Origene PTEN cDNA (SC119965), FLAG-Crm1 [18] (Addgene plasmid #17647), FLAG-PTEN [19] (Addgene plasmid #22231), PTEN-E4 [12] (Addgene plasmid #11171), PTEN-A4 [12] (Addgene plasmid #10753), HA-PTEN-NLS [20](Addgene plasmid #10933) and HA-PTEN-wt [20] (Addgene plasmid #10750). Wild type PTEN cDNA from Origene was used by Genewiz (La Jolla, CA) as a template for site-directed mutagenesis to mutate the codons encoding Leucine residues at amino acids 334, 336 and 338 to Alanine residues. The resulting construct is referred to as PTEN-AAA.

### Preparation of tumor tissue microarray

27 formalin-fixed paraffin-embedded (FFPE) prostate carcinomas were obtained from the Hollings Cancer Center Tissue Biorepository (Medical University of South, Carolina). These tissues were obtained in accordance with an Institutional Review Board approved protocol (426). One-millimeter tissue cylinders were punched from representative tumor areas of a “donor” tissue block and brought into different recipient paraffin blocks each containing 27 individual samples. Three tissue cores were sampled from each tumor, and one core was sampled from patient matched adjacent normal tissue. Four-micrometer thick sections of the TMA were cut and processed for immunohistochemistry.

### Immunohistochemistry

FFPE sections were deparaffinized in xylene, rehydrated in alcohol, and processed for pretreatment as follows: The sections were incubated with target retrieval solution (Dako, Glostrup, Denmark) in a steamer (Rival, CA, USA) for 45 minutes and then 3% hydrogen peroxide solution for 10 minutes and protein block (Dako, Glostrup, Denmark) for 20 minutes at room temperature. Primary antibody incubation overnight in a humid chamber at 4°C followed by biotinylated secondary antibody (Vector, CA, USA) for 30 minutes and ABC reagent (Vector, CA, USA) for 30 minutes. Immunocomplexes of horseradish peroxidase were visualized by DAB (Dako, Glostrup, Denmark) reaction, and sections were counterstained with hematoxylin before mounting. Immunoreactivity was scored using a semiquantitative system combining intensity of staining(0-3) and percentage of cells staining positive(0-3). Separate scores were generated for PTEN staining within the nucleus and the cytoplasm.

### Confocal Microscopy

Cells grown on 8-well chamber slides (Becton-Dickinson, Franklin Lakes, NY) were fixed in 3.7% paraformaldehyde, permeabilized in methanol, blocked with 1.5% bovine serum albumin in PBS, and incubated with PTEN primary antibody in 1% BSA/PBS. Bound primary antibodies were detected with Alexa Fluor 555-conjugated goat anti-rabbit secondary antibodies (Invitrogen). Nuclei were stained by incubation with 1:5000 diluted ToPro3-Iodide for 15 minutes. Confocal microscopy was performed using a Leica Laser Scanning Confocal Microscope maintained by the Medical University of South Carolina Hollings Cancer Center Cell and Molecular Imaging Core (1P30 CA138313-01). For each treatment, a minimum of 100 cells were evaluated visually for presence of nuclear PTEN by an evaluator blinded to the treatment group. NIH ImageJ software was used to measure the cytoplasmic and nuclear PTEN stain intensities and the N/C ratios for each treatment were determined.

### Immunoprecipitation

Cells transfected with either FLAG-Crm1 [18] (Addgene plasmid #17647) or FLAG-PTEN [19] (Addgene plasmid #22231) were used for Immunoprecipitation with the Sigma FLAG IP kit in accordance with manufacturer instructions. Briefly, cells were washed twice with ice cold PBS then lysed by gentle shaking with lysis buffer at 4°C for 20 minutes. Cells were scraped and collected into centrifuge tubes, and cellular debris was pelleted by centrifugation at 12,000 g for 12 minutes. Cell lysates were used to suspend anti-FLAG M2 affinity beads. Following overnight rotation at 4°C, beads were pelleted and washed several times before the bound protein was liberated with boiling for 5 minutes in 2X sample buffer. After pelleting the beads, 15µL of the supernatant was analyzed by immunoblotting as described above.

### Cellular Fractionation

Nuclear and cytoplasmic fractions were obtained using the REAP method [21], with slight modification. Briefly, cells were washed twice with ice cold PBS before mechanical disruption of cytoplasmic membranes by tituration (5X with P-1000 pipetor) with .1% NP-40 in PBS. After a 10 second spin in a table top microcentrifuge, the supernantant was reserved as the cytoplasmic fraction. Following an additional brief tituration (2X with P-1000 pipetor) and a 10 second centrifugation, the supernatant was removed and the pellet was resuspended in 30µL RIPA buffer. After 30 minutes of incubation on ice with aggressive vortexing every 10 minutes, nuclear debris was removed by centrifuging at 20,000 x g for 20 minutes. The supernatant is the nuclear fraction.

### Adenovirus infection

AC cDNA was purchased from Origene (Rockville, MD) and Ad-AC (adenovirus expressing AC and GFP) and Ad-GFP were developed by Vector Biolabs (Philadelphia, PA). Ad-PTEN was purchased from Vector Biolabs. The short hairpin sequence (5’- ccgggctgttattgacagcgatatactgagtatatcgctgtcaataacagcttttt -3’) obtained from Open Biosystems (Huntsville, AL) was validated and developed into an adenoviral delivery vector (Ad-shAC) by Vector Biolabs. The HA-PTEN-NLS construct obtained from Addgene was developed into an adenoviral delivery vector by Vector Biolabs by cloning the PTEN-NLS sequence without the HA tag into an adenovirus construct. Cells were infected in suspension with the indicated MOI of the indicated adenovirus in complete medium.

### Western blotting

Cell lysates were prepared and analyzed as previously described [22], using the following antibodies: pAkt (#4060), total-Akt (#4691) p-P70S6K (#9234), P70S6K (#9202), PTEN (#9559), p-PTEN S380(#9551), p-PTEN S380/Thr382/383 (#9554) Histone-H3 (#4499), β-Tubulin (#2128) DYKDDDDK (FLAG) Tag (#2044) (Cell Signaling Technologies, Danvers, MA), AC (BD Transduction #612302), GAPDH (G-9), and Lamin B (C-20) (Santa Cruz, Santa Cruz, CA).

### Cell viability assays

5000 cells per well were infected with Ad-AC or Ad-GFP and plated in 96-well plates. After overnight attachment, medium containing the indicated compound was added. Cells were treated with a broad dose range of Docetaxel from 100 nM to .1 nM, doses encompassing effecting little to complete cell death. After 48 hours, the Promega CellTiter 96® AQueous One Solution Cell Proliferation Assay (MTS) was used to approximate the number of viable cells. Prism v4 was used to determine the EC50 (concentration at which half of the cells are viable).

### Propidium Iodide staining

Cells were trypsinized and washed twice with cold PBS prior to suspension in 100µL PBS followed by 3 ml of -20°C 70% EtOH. After a minimum of one hour incubation at 4°C, cells were washed twice with 2 ml PBS and again resuspended in 100µL PBS. Cells were incubated or one hour with RNase A at a final concentration of 500µg/ml. 20µL of stock solution of propidium iodide (1 mg/ml) was added to a final concentration of 50µg/ml and incubated in the dark at 4°C for 30 minutes prior to analysis for DNA content by flow cytometry with software rendering by ModFit LT 4.0 (Verity Software House).

### Proliferation

2000 cells were plated per well in 24-well plates. On the indicated day (day 0 being the day after plating), one plate was fixed in 3.7% formalin and stained with crystal violet. Cell number was determined by counting a minimum of 5 randomly selected microscopic fields (10X objective).

### Animal studies

For establishment of xenografts, cells were infected at MOI 25 with each of the indicated adenoviruses in suspension at a density of 1x10^6^ cells per mL in serum free medium containing 1% BSA for 4 hours prior to preparation for engraftment by suspension in 1:1 RPMI 1640: Matrigel HC admixture (0.08 mL per injection). Pathogen-free four-week-old female Athymic NCr-nu/nu mice were purchased from the NCI, Frederick Cancer Research Center (Frederick, MD, USA). Animals were monitored for formation of palpable tumors weekly. The mice were maintained under standard conditions according to the institutional guidelines for animal care. All animal experiments were approved by the Committee for the Care and Use of Laboratory Animals of Medical University of SC.

### Statistical Analysis

Independent experiments were performed a minimum of three times. Statistical analyses on experiments performed in triplicate were performed by unpaired one-tailed student’s *t* test, one way ANOVA with Bonferroni correction using Prism (version 4.0) from GraphPad, or Fisher’s Exact Test. *p<0.05 was considered significant. Time to tumor formation was analyzed via Kaplan-Meier methods. Mice that did not develop tumors were censored at 42 days. The log-rank test was used to compare distributions of time to tumor formation across groups for all pairs of groups.

## Results

### Acid ceramidase correlates with loss of nuclear PTEN in prostate adenocarcinoma

**Figure 1:**
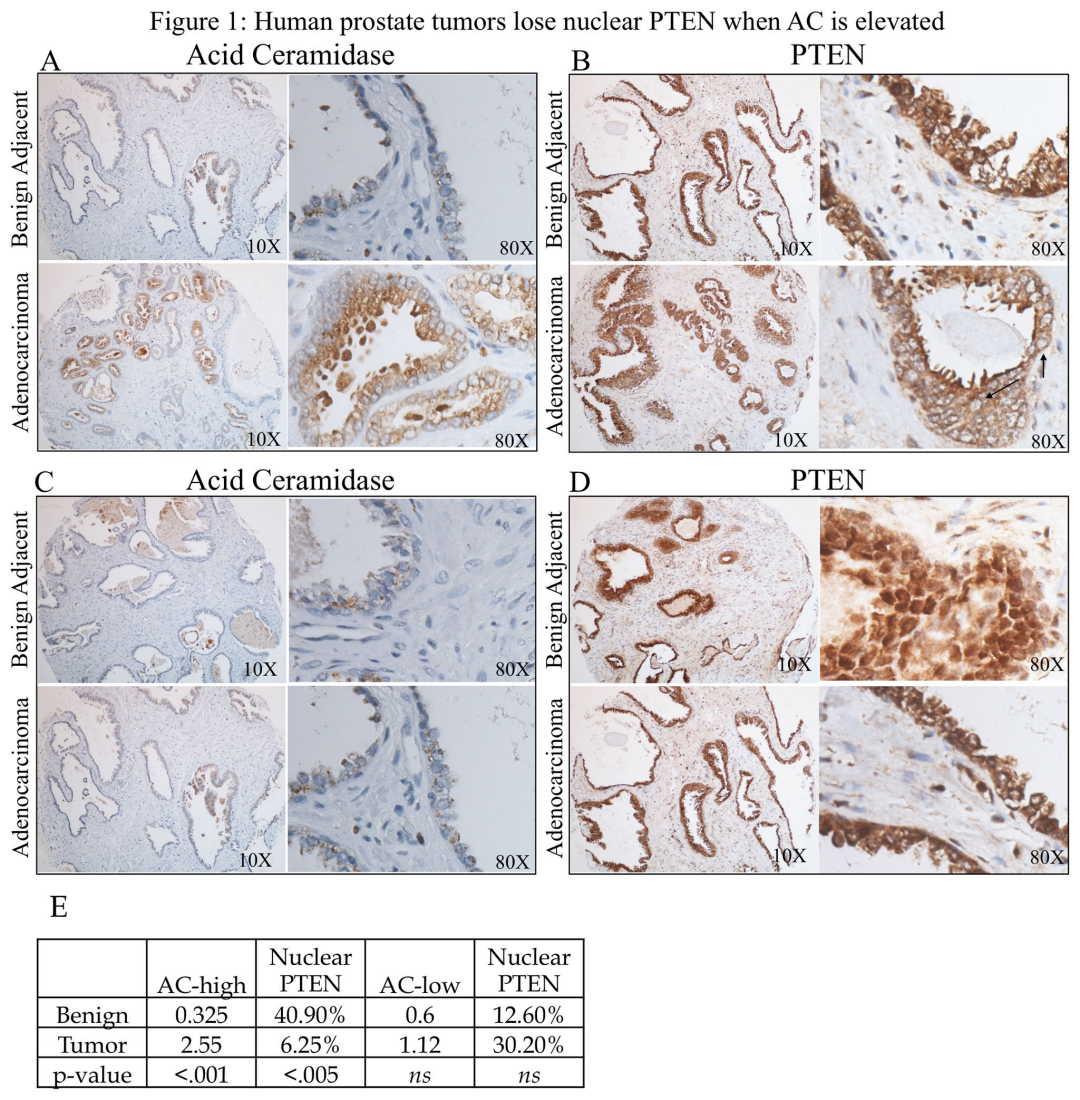
Human prostate tissues lose nuclear PTEN when AC is elevated. (*a*-*d*) A 27 patient tissue microarray with patient-matched prostate adenocarcinoma and adjacent benign tissue was immunostained for AC and PTEN. Staining intensities for AC, nuclear PTEN, and cytoplasmic PTEN were evaluated by a blinded pathologist. The arrows in (*b*) are examples of nuclei devoid of PTEN. In patients whose cancer tissue had elevated AC than their benign tissue (*a*), there was also a decrease in the amount of nuclear PTEN, p<.05 (b). Patients whose AC did not increase in their tumor tissue (*c*) did not have a decrease in nuclear PTEN (*d*). AC pathology scores and nuclear PTEN percentage for the AC-high and AC-low patient groups are organized in (*e*).

Using a tissue microarray (TMA) made up of prostate adenocarcinoma and patient-matched benign adjacent biopsy cores from 27 prostate cancer patients, we determined that in the patients whose tumor AC immunohistochemistry (IHC) staining was elevated compared their benign AC score (benign AC score: .325, adenocarcinoma AC score: 2.55, p<.001) (Figure 1A), the percentage of PTEN in the nuclei of the specimens (100*nuclear PTEN score/(cytoplasmic + nuclear PTEN score) was decreased in adenocarcinoma tissue (benign nuclear PTEN: 40.9%, adenocarcinoma nuclear PTEN: 6.25%, p<.05) (Figure 1B). Conversely, in patients whose tumor AC staining was not elevated compared to their benign tissue (Figure 1C) no decrease in the percentage of nuclear PTEN was observed (Figure 1D). The AC scores and nuclear PTEN % are displayed in table form (Figure 1E).

### Acid ceramidase causes S1P-dependent loss of nuclear PTEN

**Figure 2:**
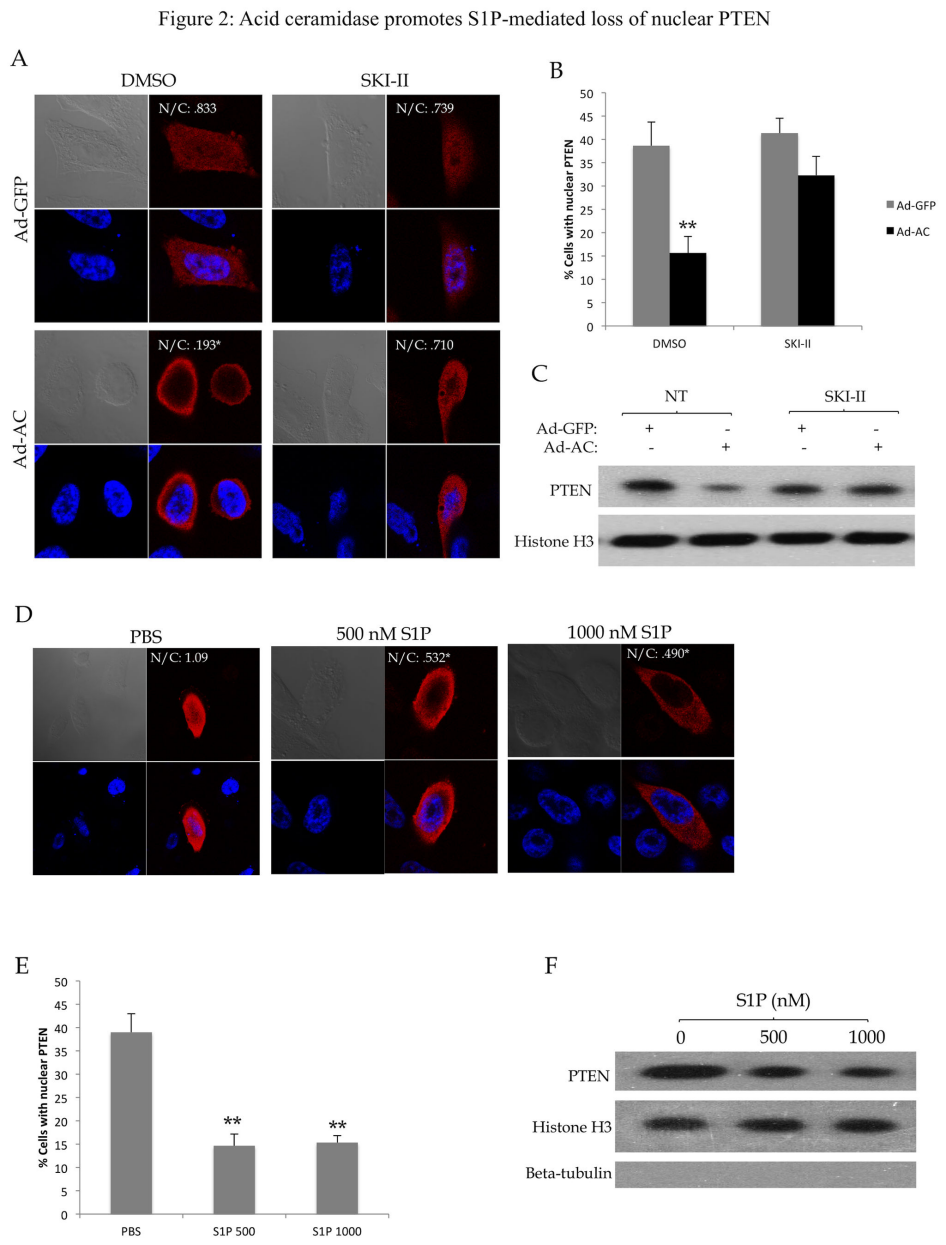
Acid ceramidase promotes S1P-mediated loss of nuclear PTEN. PPC1 cells transfected with WT-PTEN were infected with Ad-GFP or Ad-AC for 48 hours in the presence of DMSO (no treatment; NT) or the sphingosine kinase inhibitor SKI-II for 24 hours. A) Cells were immunostained for PTEN (red) and nuclei (blue). Nuclear (N) and cytoplasmic (C) PTEN staining intensity were measured for all cells in a given treatment using ImageJ. N/C indicates the nuclear PTEN to cytoplasmic PTEN ratio. B) The percentage of cells from (A) which had nuclear PTEN in each treatment. C) Nuclear fractions from the indicated treatments were isolated and evaluated for presence of PTEN with Histone H3 as a nuclear loading control and absence of β-tubulin to indicate purity of the nuclear sample. D) PPC1 cells transfected with WT-PTEN were treated with the indicated dose of S1P or PBS for 2 hours prior to fixation and immunostaining for PTEN (red) and nuclei (blue). E) The percentage of cells from (D) which had nuclear PTEN. F) Nuclear fractions from the indicated treatments were isolated and evaluated for presence of PTEN with Histone H3 as a nuclear loading control and absence of β-tubulin to indicate purity of the nuclear sample. One way ANOVA with Bonferroni correction, *p<.05, **p<.01.

PPC1 cells transfected with wild type PTEN were infected with either Ad-GFP or Ad-AC. Figure S1 is provided as a demonstration of the degree of AC expression that is enforced using our adenoviral transfection approach which can be compared to the degree of AC overexpression found in human prostate tumors in our previous study [23]. Immunofluorescence to visualize PTEN localization revealed that expression of AC significantly reduced the number of cells that had PTEN in the nucleus, but not in the presence of the sphingosine kinase inhibitor SKI-II. Representative cells are shown in Figure 2A, and quantified in Figure 2B. Nuclei from cells infected with Ad-GFP or Ad-AC were isolated and evaluated for nuclear PTEN by western blotting (Figure 2C). Nuclear PTEN was reduced in Ad-AC infected cells, but not in the presence of the sphingosine kinase (SphK) inhibitor SKI-II, suggesting that AC promotes a reduction in nuclear PTEN by a SphK-dependent mechanism. Whole cell lysates from this experiment are shown in Figure S2A. In order to maintain consistency throughout the study, we utilized PPC1 cells, which are PTEN null, transfected with PTEN constructs. This allows evaluation of PTEN and PTEN mutant localization without the effects of endogenous PTEN. In order to verify that this phenomenon occurs with endogenous PTEN, key results were confirmed in DU145 cells. Stable (Figure S3A-B) and transient (Figure S3C-D) expression of AC promoted reduction in nuclear PTEN that was blocked by SKI-II, similar to the results obtained in transfected PPC1 cells. To determine whether exogenous S1P could mimic the effects of AC expression, we treated cells with 500 or 1000 nM S1P. Treatment of cells with S1P recapitulated this phenomenon in PTEN-transfected PPC1 (Figure 2D-F, Figure S2B) as well as in DU145 cells (Figure S3E-H), indicating that S1P promotes a reduction in nuclear PTEN in prostate cancer cells.

### S1P promotes Akt-dependent loss of nuclear PTEN

**Figure 3:**
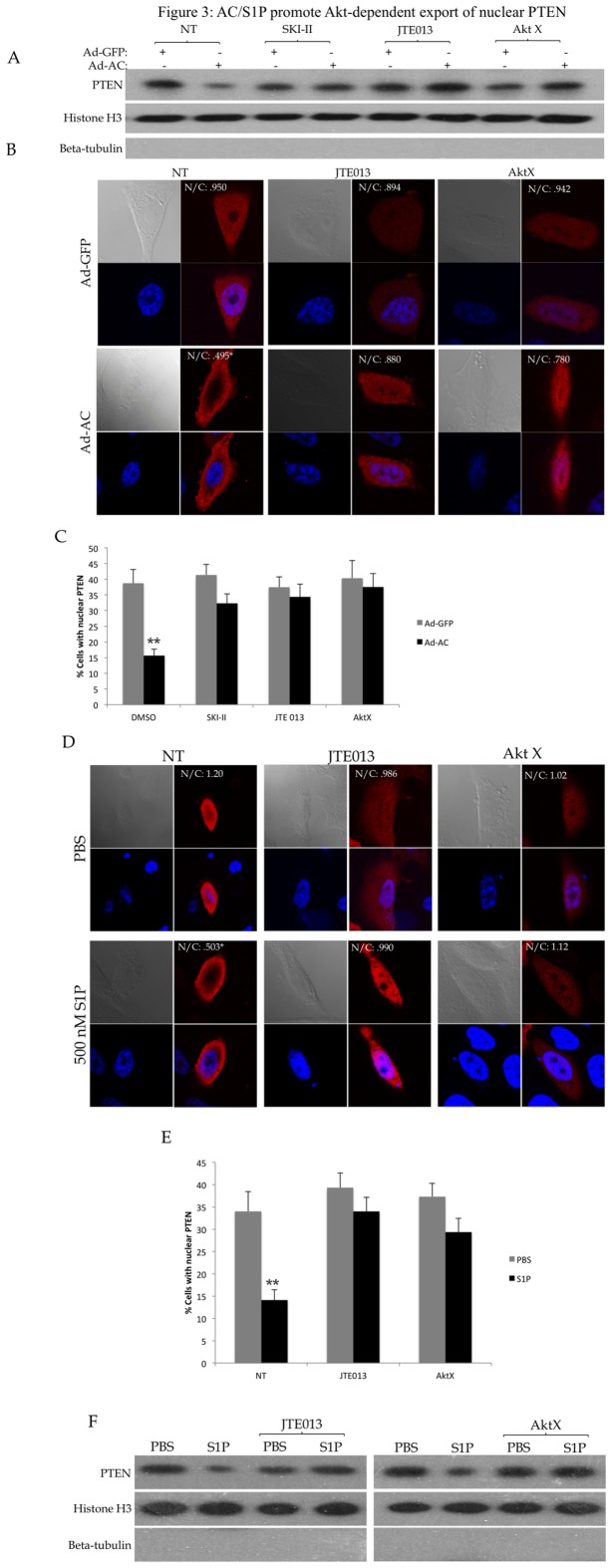
AC/S1P promote Akt-dependent export of nuclear PTEN. PPC1 cells were transfected with WT-PTEN were infected with Ad-GFP or Ad-AC for 48 hours in the presence of DMSO (NT) or the indicated compounds for 24 hours. A) Nuclear fractions from the indicated treatments were isolated and evaluated for presence of PTEN with Histone H3 as a nuclear loading control and absence of β-tubulin to indicate purity of the nuclear sample. B) Cells were immunostained for PTEN (red) and nuclei (blue). C) The percentage of cells from (B) which had nuclear PTEN in each treatment. D) PPC1 cells transfected with WT-PTEN were treated with 1µM JTE013 or 5µM AktX for 24 hours prior to treatment with the indicated dose of S1P or PBS for 2 hours followed by fixation and immunostaining for PTEN (red) and nuclei (blue). E) The percentage of cells from (D) which had nuclear PTEN. F) Nuclear fractions from the indicated treatments were isolated and evaluated for presence of PTEN with Histone H3 as a nuclear loading control and absence of β-tubulin to indicate purity of the nuclear sample. One way ANOVA with Bonferroni correction, *p<.05**p<.01.

Recent studies from our lab have shown that AC causes activation of Akt through S1P receptor 2 (S1PR2) in prostate cancer cells. To determine whether AC-induced Akt activation mediates loss of nuclear PTEN, we expressed AC in PPC1 cells and analyzed changes in PTEN localization using nuclear fractionation (Figure 3A) and confocal microscopy (Figure 3B-C). Antagonism of S1PR2 with JTE013 abolished AC-mediated nuclear PTEN loss, as did inhibition of Akt with the inhibitor AktX, indicating that AC-induced Akt activation promotes loss of nuclear PTEN. Treatment of cells with exogenous S1P in the presence of JTE013 and AktX demonstrated that S1P, through S1PR2 and activation of Akt, promotes loss of nuclear PTEN (Figure 3D-F). Inhibition of S1PR2 or Akt prevented S1P-mediated nuclear PTEN loss in DU145 as well (Figure S4).

### S1P mediates Crm1-dependent export of nuclear PTEN

**Figure 4:**
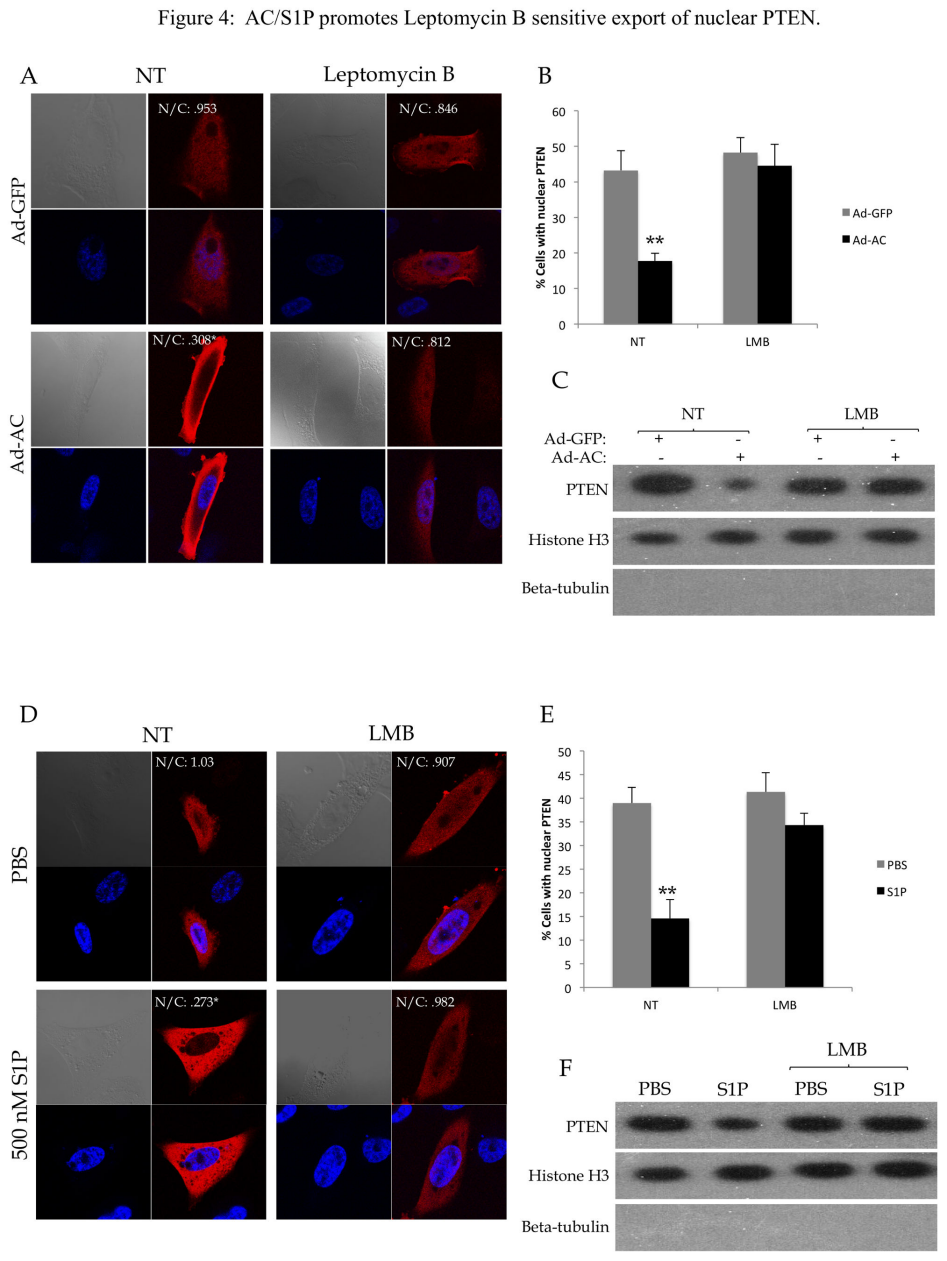
AC/S1P promotes Leptomycin B sensitive export of nuclear PTEN. PPC1 cells transfected with WT-PTEN were infected with Ad-GFP or Ad-AC for 48 hours in the presence of EtOH (NT) or 100 nM Leptomycin B (LMB) for 24 hours. A) Cells were immunostained for PTEN (red) and nuclei (blue). B) The percentage of cells from (A) which had nuclear PTEN in each treatment. C) Nuclear fractions from the indicated treatments were isolated and evaluated for presence of PTEN with Histone H3 as a nuclear loading control and absence of β-tubulin to indicate purity of the nuclear sample. D) PPC1 cells transfected with WT-PTEN were treated with 100nM LMB for 24 hours and 500nM S1P or PBS for 2 hours prior to fixation and immunostaining for PTEN (red) and nuclei (blue). E) The percentage of cells from (D) which had nuclear PTEN. F) Nuclear fractions from the indicated treatments were isolated and evaluated for presence of PTEN with Histone H3 as a nuclear loading control and absence of β-tubulin to indicate purity of the nuclear sample. One way ANOVA with Bonferroni correction, *p<.05**p<.01.

**Figure 5:**
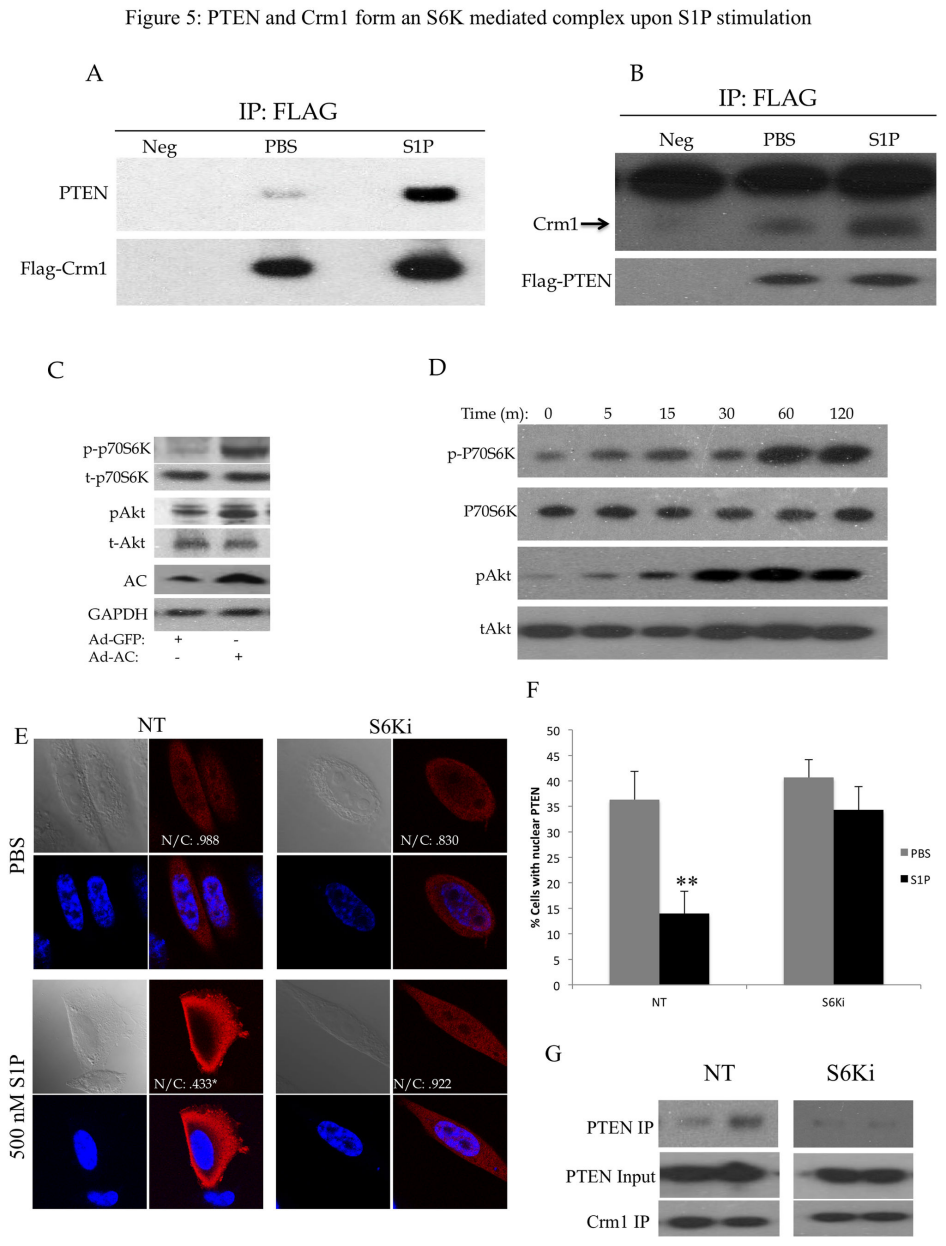
PTEN and Crm1 form an S6K mediated complex upon S1P stimulation. A) PPC1 cells were transfected with WT-PTEN and FLAG-Crm1. Cells were collected after 2 hours stimulation with 500nM S1P or PBS and submitted to immunoprecipitation of the FLAG-Crm1 protein. The negative control (Neg) indicates lysate from cells not transfected with FLAG-Crm1. B) PPC1 cells were transfected with FLAG-PTEN and collected after 2 hour stimulation with 500nM S1P or PBS and submitted to immunoprecipitation of the FLAG-PTEN protein. The negative control (Neg) indicates lysate from cells not transfected with FLAG-PTEN. C) PPC1 cells were infected with Ad-AC or Ad-GFP and analyzed for S6K phosphorylation. D) PPC1 cells were treated with 500nM S1P for 2 hours and analyzed for S6K phosphorylation. E) PPC1 cells transfected with WT-PTEN were treated with water (NT) or 2.5 µM S6K1 inhibitor DG2 (S6Ki) for 24 hours then stimulated with 500 nM S1P for 2 hours prior to immunostaining for PTEN (red) and nuclei (blue). F) The percentage of cells from (E) which had nuclear PTEN in each treatment. G) PPC1 cells were transfected with WT-PTEN and FLAG-Crm1 and treated for 24 hours with 2.5 µM S6Ki. Cells were collected after 2 hour stimulation with 500nM S1P or PBS and submitted to immunoprecipitation of the FLAG-Crm1 protein. One way ANOVA with Bonferroni correction, *p<.05**p<.01.

To investigate the mechanism of S1P-mediated nuclear loss of PTEN, we used the Crm1 inhibitor Leptomycin B (LMB) to determine whether we were observing a Crm1-dependent PTEN nuclear export. Indeed, LMB abrogated AC- (Figure 4A-C) and S1P- (Figure 4D-F, Figure S5) induced nuclear PTEN loss, suggesting that we are observing activation of Crm1-mediated active export of PTEN upon AC expression or S1P stimulation. To determine whether S1P promotes association of PTEN and Crm1, we transfected cells with either FLAG-Crm1 (Figure 5A) or FLAG-PTEN (Figure 5B) and analyzed FLAG immunoprecipitates. Interestingly, stimulation of cells with S1P significantly promoted PTEN presence in FLAG-Crm1 immunoprecipitates and, reciprocally, Crm1 in FLAG-PTEN immunoprecipitates, suggesting that S1P stimulates formation of a complex between Crm1 and PTEN. Whole cell lysates from this experiment are shown in Figure S6. PTEN does not have a described nuclear export sequence (NES), however *in silico* analysis with NetNES1.1 [24] predicts a weak NES (Figure S7A). Site directed mutagenesis of three Leucine residues in the predicted NES to Alanine residues (L334A, L336A and L338A) did not impact S1P-mediated nuclear export (Figure S7B) or reduce the presence of PTEN in S1P-stimualted Crm1 immunoprecipitates (Figure S7C), suggesting that this predicted NES is not functional. We also evaluated whether phosphorylation of the C-terminus of PTEN was involved in S1P-mediated nuclear export of PTEN, as the phosphorylation status of the C-terminus has been reported to be involved with its nuclear localization [9,25]. Expression of AC did not promote C-terminus phosphorylation of PTEN (Figure S8A), and two mutant versions with Alanine or Glutamate substitutions in phosphorylation residues that were identified as regulating localization of PTEN, A4 (S380A, T382A, T383A, S385A) and E4 (S380E, T382E, T383E, S385E) were both found in S1P-stimulated Crm1 immunoprecipitates (Figure S8B). PTEN-E4 and PTEN-A4 were PTEN were equivalently exported upon S1P stimulation (Figure S8C), thus we concluded that PTEN C-terminus phosphorylation is not involved in AC/S1P induced nuclear export of PTEN.

### AC and S1P mediate Crm1-dependent export of nuclear PTEN through S6K activation

Previous reports have shown that Akt promotes PTEN nuclear export through Crm1, mediated by a physical interaction with S6K [13]. In keeping with our previous findings that AC, through S1P, promotes activation of Akt and its downstream targets, overexpression of AC (Figure 5C) and treatment with exogenous S1P (Figure 5D) promoted phosphorylation of S6K. To evaluate whether S6K-dependent nuclear export of PTEN was involved in our observations, we used the S6K1 inhibitor S6K1 Inhibitor II (here called S6Ki). The S6K inhibitor prevented S1P-mediated PTEN nuclear egress (Figure 5E-F). Moreover, utilization of S6Ki prevented the association of PTEN with Crm1 upon S1P stimulation, as noted by the absence of S1P-stimulated increase in PTEN in FLAG-Crm1 immunoprecipitates in the presence of S6Ki (Figure 5G). These findings strongly suggest that AC/S1P promote nuclear egress of PTEN through the formation of an S6K-mediated complex with Crm1, as previously described.

### Oncogenic features of AC expressing cells are absent in the presence of nuclear localized PTEN

**Figure 6:**
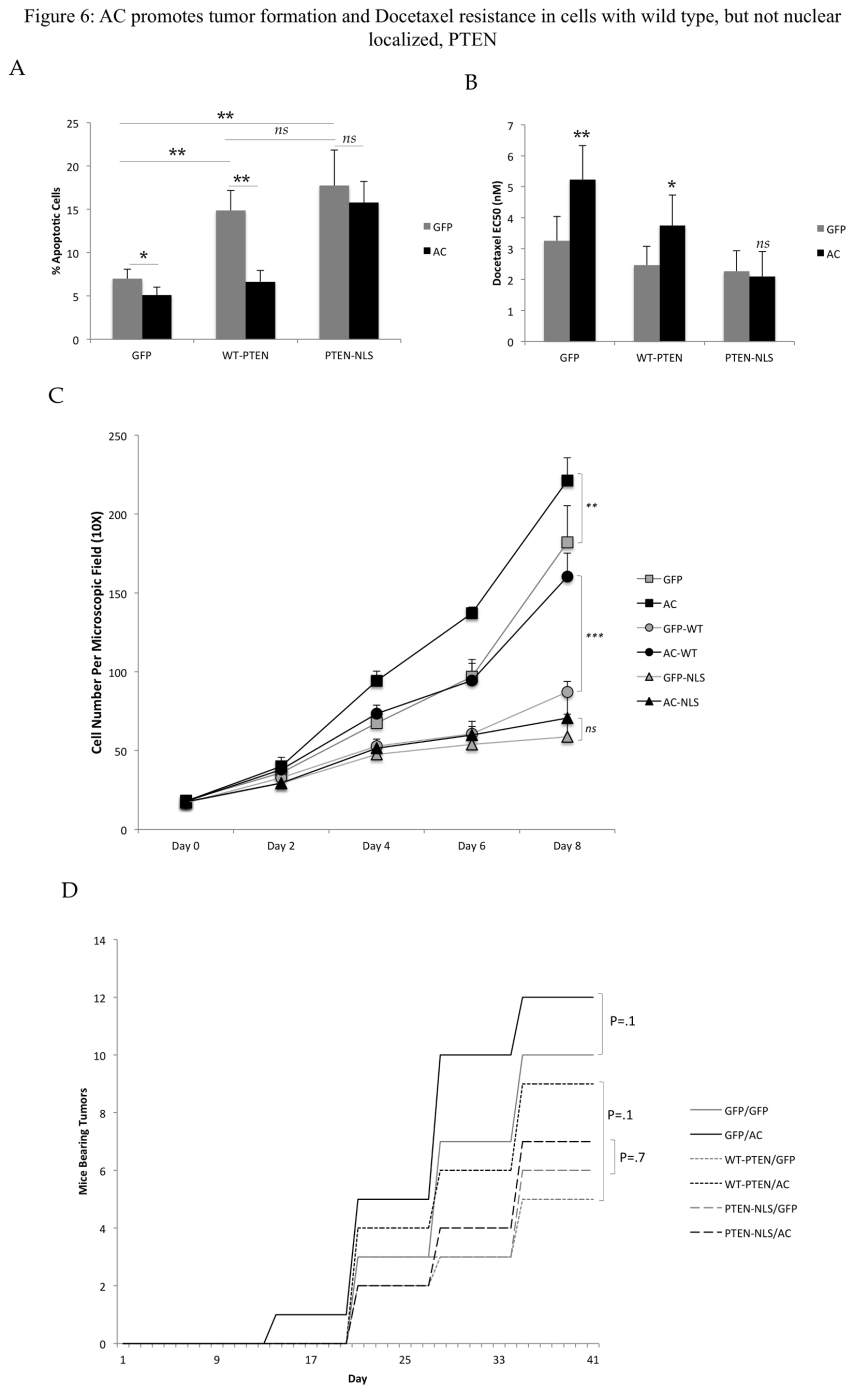
AC promotes tumor formation and Docetaxel resistance in cells with wild type, but not nuclear localized, PTEN. A-D) PPC1 cells were infected with 25 MOI Ad-GFP or Ad-AC and either 25 MOI Ad-GFP, Ad-WT-PTEN, or Ad-PTEN-NLS. A) After 24 hours plating, cells were treated with 1.5nM Docetaxel and after a further 48 hours, stained with propidium iodide and analyzed for apoptotic cells using FACS. B) After 24 hour attachment, cells were treated with a dose course (.01 to 100nM) Docetaxel and analyzed for relative cell viability using MTS assay after a further 48 hours. The EC50 was estimated using Prism 4 software. C) Cells were counted on the indicated day (day 0 being the day of plating). Student’s t-test, *p<.05, **p<.01. D) 4x106 cells were injected into the flanks of nu/nu mice and observed for 6 weeks. We monitored the mice each week for the formation of palpable tumors and graph the number of mice in the indicated treatment that had established palpable tumor at the indicated day.

Nuclear PTEN has been found to promote apoptosis and suppress cell proliferation. Therefore, we set out to determine whether the ability of AC to promote nuclear egress of PTEN was functionally important for these phenotypes. We co-infected PPC1 cells with Ad-GFP or Ad-AC and Ad-GFP, Ad-WT-PTEN, or Ad-PTEN-NLS. AC promoted nuclear egress of WT-PTEN, but not NLS-PTEN, which remained confined to the nucleus in all cells (Figure S9A). Moreover, PTEN-NLS and WT-PTEN are expressed at similar levels (Figure S9B) Treatment of cells with 1.5 nM Docetaxel induced apoptosis in cells expressing both WT-PTEN and PTEN-NLS more strongly than cells not expressing PTEN (Figure 6A), consistent with the role of PTEN in potentiating apoptotic stimuli. Interestingly, while Ad-AC was able to suppress Docetaxel induced apoptosis in Ad-GFP and Ad-WT-PTEN infected cells, Ad-PTEN-NLS infected cells were not protected from apoptosis by Ad-AC. Moreover, the EC50s of these treatments to Docetaxel were increased by Ad-AC, indicating desensitization or protection from cell death due to Docetaxel, however Ad-PTEN-NLS infected cells had no change in EC50 when AC was expressed (Figure 6B). These results suggest that AC is able to protect cells from chemotherapy-induced apoptosis and cell death when it is able to promote nuclear egress of PTEN, but not when nuclear expression of PTEN is enforced. In a study of cell proliferation, Ad-GFP and Ad-WT-PTEN infected cells expressing AC proliferated more rapidly than their Ad-GFP infected controls (Figure 6C). Interestingly, while the overall rate of proliferation in WT-PTEN expressing cells was slower, Ad-AC promoted proliferation more robustly (1.84 fold at day 8) compared to cells expressing no PTEN (1.2 fold at day 8), suggesting that while AC can promote proliferation in the presence and absence of PTEN, it exerts a more powerful influence in PTEN expressing cells. Cells expressing PTEN-NLS proliferated the least of all treatments, and Ad-AC provided no advantage in proliferation, further suggesting that the ability of AC to promote proliferation depends upon its ability to promote nuclear egress of PTEN. In a study of the ability of AC to promote tumor engraftment in a xenograft study, we found that AC trended towards promotion of formation of tumors more rapidly in cells expressing GFP (control) or a WT-PTEN construct (p=0.1), but not in cells expressing NLS-PTEN (p=0.7) (Figure 6D).

## Discussion

Once thought to be an exclusively cytoplasmic protein, PTEN has been found in the nuclei of numerous cell types. Interestingly, studies in breast tissue [26], thyroid tissue [27], and pancreatic tissue [28] have all found that nuclear PTEN is generally found in benign or resting cells but tends to be lost in more aggressive cancer cells. Because PTEN is now well known to be a tumor suppressor in the nucleus in addition to its well-appreciated role in cytoplasmic tumor suppression, determining how PTEN gets in and out of the nucleus is of great interest. A number of studies describe mechanisms of PTEN nuclear import [7,9,11,12], however very little information is known about export of PTEN from the nuclear compartment. We initially observed that cells overexpressing AC seemed to have a reduced amount of nuclear PTEN compared to controls, which led us to investigate the mechanism behind this observation. Having recently discovered that AC promotes activation of Akt through S1P receptor 2-mediated stimulation of PI3K [14], we sought to determine whether AC-induced reduction of nuclear PTEN is mediated by S1P-induced activation of Akt. By inhibiting sphingosine kinase, S1PR2, and Akt in AC-overexpressing cells, we found that the previously described pathway of AC-induced Akt activation was indeed necessary for AC to promote nuclear egress of PTEN. The essential role of this pathway was confirmed using S1PR2 and Akt inhibition in cells stimulated with exogenous S1P.

Having found that AC-mediated Akt activation promoted loss of nuclear PTEN, we were interested in determining what mechanisms of PTEN nuclear export might be responsible for the phenomenon we observed. Our mutation of an *in silico* predicted nuclear export sequence (NES) and multiple C-terminus phosphorylation sites did not support classical direct nuclear export or alteration in PTEN C-terminus phosphorylation as contributory to AC/S1P-mediated nuclear export of PTEN [12]. Alfred Yung’s group has found evidence that PTEN moves from the nucleus to the cytoplasm in response to Akt activation through mTOR-mediated activation of S6K [13]. In that study, inhibition of PI3K, mTOR, and S6K1 all prevented export of PTEN. They concluded that this export was dependent on Crm1, as the inhibitor Leptomycin B prevented export of PTEN from the nucleus. Overall, they presented compelling evidence of a negative feedback loop through which activation of Akt is suppressed by recruiting a nuclear reservoir of PTEN into the cytoplasm. Because we observed AC-induced, Akt-dependent PTEN nuclear egress, we sought to determine whether the previously discovered Crm1-mediated S6K dependent export was occurring in our system. Indeed, we found that not only does Leptomycin B suppress AC and S1P mediated export of nuclear PTEN, as found in the Yung study, but also the novel observation that S1P stimulation promotes formation of a physical complex between PTEN and Crm1. Similar to the Yung study, inhibition of S6K prevented export of nuclear PTEN and abolished S1P-mediated formation of the Crm1-PTEN complex, suggesting that the previously described mechanism is active in our observed phenomenon. The Yung study specifically outlined Akt-mediated export of PTEN at the G1/S transition. Our results do not implicate a cell-cycle dependent event as we observed a reduction in nuclear PTEN intensity in 100% of cells following S1P treatment, and synchronization in G1 by serum starvation did not affect the localization of PTEN or the impact of AC/S1P on the percentage of intensity of nuclear PTEN (data not shown). The discrepancy is likely due to the different cell types used between our study and Yung’s, in which the majority of the data regarding the G1/S transition was gathered in fibroblasts compared to our study, which uses epithelial prostate carcinoma cells in which derangements in cell cycle processes are severe.

While identifying the mechanism by which AC promoted nuclear loss of PTEN was a chief goal of this study, we were also interested in whether PTEN translocation affects a relevant disease state. AC is overexpressed in the majority of prostate tumors at the mRNA [29] and protein [23] levels, and years of study by our group have shown that AC promotes oncogenic phenotypes in prostate cancer by promoting resistance to chemotherapy [16] and radiotherapy [22] and promoting cell proliferation and xenograft growth [16]. Thus, AC overexpression is a relevant model in which to investigate whether nuclear export of PTEN is an impactful event on the behavior of prostate cancer. To establish this, we evaluated expression of AC and nuclear and cytoplasmic expression of PTEN in a human prostate TMA which contains 27 patient matched adenocarcinoma and benign adjacent tissues, allowing us to evaluate molecular alterations that occur in an individual patient’s diseased tissue. In this analysis, we found that in patients whose cancer tissue had elevated AC expression compared to their benign tissue also experienced a loss of nuclear PTEN in the benign to cancer transition. Patients whose tumors did not upregulate AC did not lose nuclear PTEN. This mirrors observations in melanoma, colon cancer, and others in which nuclear PTEN was more prevalent in benign tissue than in cancer [5], with the added implication that AC promotes nuclear egress of PTEN during the development of human prostate cancer. These observations that nuclear PTEN loss may be a consequence of AC overexpression are interesting as nuclear PTEN loss has been found to be a negative prognostic indicator in multiple cancer types.

Functionally, we investigated two of the processes that nuclear PTEN has been found to mediate: apoptosis and proliferation. While some studies have shown that nuclear PTEN does not mediate apoptosis [30], nuclear PTEN is known to regulate p53 acetylation [31,32] and promote apoptosis in response to TNF alpha and doxorubin [9]. To induce apoptosis, we used the standard of care therapy for hormone refractory prostate cancer, Docetaxel, finding that AC expression rescued PPC1 cells expressing wild type PTEN from apoptosis with a concomitant increase in the EC50 of Docetaxel in these cells. This observation is largely consistent with our previous report that AC expression in DU145 cells, which bear wild type PTEN, promotes resistance to taxanes [16]. In contrast, cells expressing nuclear localized PTEN were not protected from Docetaxel by expression of AC, which promoted no change in percentage of apoptotic cells or EC50. This observation identifies a potential mechanism by which active reduction in nuclear PTEN may promote escape from apoptosis in response to chemotherapy and potentially other therapeutics. The Pandolfi group has recently shown impressive evidence that nuclear PTEN suppresses the APC/C (anaphase-promoting complex/cyclosome), which opposes several cell-cycle promoting proteins by promoting their ubiquitin-mediated degradation [4]. This study provides strong mechanistic and functional evidence that nuclear PTEN opposes cell proliferation. Interestingly, expression of AC in cells bearing wild type PTEN promoted cell proliferation 53% more than cells bearing no PTEN, suggesting that while AC promotes cell proliferation in the absence of PTEN, the presence of PTEN allows a more prominent effect, suggesting that the interaction of AC with PTEN is a factor in its ability to promote cell proliferation. It is worth noting that the PTEN-independent promotion of proliferation by AC is not surprising, as Akt has several well-known roles in promoting cell proliferation. In contrast, expressing AC in cells with enforced nuclear expression of PTEN had no impact on cell proliferation, again illustrating that the ability of AC to promote nuclear export of PTEN is an important part of its suppression of oncogenic properties in prostate cancer cells. To test whether the impact of AC on PTEN was important in vivo, we found that while both PTEN-NLS and wild type PTEN suppressed xenoengraftment, AC trended towards promotion of tumor formation only in cells bearing no PTEN or WT-PTEN, although these results were not statistically significant (p=0.1). Consistent with sensitivity to Docetaxel and proliferation, AC was unable to promote tumor formation in cells bearing PTEN-NLS (p=0.7). The functional studies performed in this work present evidence that AC promotes cell proliferation, resistance to therapy, and potentially tumor formation in part through its ability to cause translocation of PTEN out of the nucleus, effectively promoting nuclear insufficiency of PTEN tumor suppression.

## Conclusion

In this study, we conclude that the Akt activation caused by AC overexpression promotes nuclear export of PTEN in prostate cancer. This phenomenon is dependent upon S1P-mediated activation of Akt which further activates S6K to promote a complex formation between PTEN and the master nuclear exporter Crm1. In human tissues, we found that upregulation of AC during the benign to tumor progression is accompanied by a loss of PTEN in the nucleus. Functional analysis revealed that the ability of AC to promote nuclear egress of PTEN was important for its promotion of proliferation, resistance to standard chemotherapy, and potentially xenoengraftment. This study provides evidence that AC, which is overexpressed in most prostate cancers, exerts its oncogenic functions in part through promoting insufficiency of PTEN tumor suppression in the nucleus.

## Supporting Information

Figure S1 **PPC1 cells were infected with Ad-AC at MOI ranging from 0 to 50, or Ad-GFP at MOI 50 and probed for expression of AC.** NIH ImageJ was used to measure band densitometries and generate AC/Actin ratios, which were normalized to Ad-GFP MOI 50.
(TIF)

Figure S2 PPC1 cells transfected with WT-PTEN were infected with Ad-GFP or Ad-AC for 48 hours in the presence of DMSO (no treatment; NT) or the sphingosine kinase inhibitor SKI-II for 24 hours (A). Whole cell lysates were analyzed by immunoblotting. (B) PPC1 cells transfected with WT-PTEN were treated with the indicated dose of S1P or PBS for 2 hours. Whole cell lysates were analyzed by immunoblotting.
(TIF)

Figure S3 **Nuclear fractions (A) and whole cell lysate (B) of DU145 cells stably expressing AC (AC-EGFP and empty vector (EGFP) were collected and analyzed by western blotting.** Nuclear fractions (C) and whole cell lysates (D) of DU145 cells infected with Ad-GFP or Ad-AC were collected and analyzed by western blotting. E-F) DU145 cells were infected with Ad-GFP or Ad-AC for 48 hours and treated with SKI-II for 24 hours prior to isolation of nuclear fractions (E) and whole cell lysates (F) and western blot analysis.G-H) DU145 cells were stimulated with 500 nM S1P for 2 hours prior to isolation of nuclear fractions (G) and whole cell lysates (H).
(TIF)

Figure S4 **DU145 cells were treated with A) 1µM JTE013 or DMSO (NT) or B) 5µM AktX or water (NT) for 24 hours prior to stimulation with 500 nM S1P or PBS (NT) for 2 hours.** Nuclear fractions were analyzed by western blotting.
(TIF)

Figure S5 **DU145 cells were treated with the indicated concentration of Leptomycin B for 24 hours prior to stimulation with 500 nM S1P for 2 hours.** Nuclear fractions were analyzed by western blotting.
(TIF)

Figure S6 PPC1 cells were transfected with WT-PTEN and FLAG-Crm1 (A). Cells were collected after 2 hours stimulation with 500nM S1P or PBS. The negative control (Neg) indicates lysate from cells not transfected with FLAG-Crm1. (B) PPC1 cells were transfected with FLAG-PTEN and collected after 2 hour stimulation with 500nM S1P or PBS. The negative control (Neg) indicates lysate from cells not transfected with FLAG-PTEN.
(TIF)

Figure S7 The amino acid sequence of PTEN was analyzed by NetNES1.1 for potential nuclear export signals (A). The identified sequence was mutated (LLL to AAA). (B) WT-PTEN and PTEN-AAA were transfected into PPC1 cells prior to stimulation with 500 nM S1P. Bars indicate the percentage of cells with PTEN in the nucleus. C) PPC1 cells were transfected with FLAG-Crm1 and either WT-PTEN or PTEN-AAA. After 2 hours stimulation with 500 nM S1P, cell lysates were immunoprecipitated with anti- FLAG beads. Student’s t-test, **p<.01.
(TIF)

Figure S8 DU145 cells were infected with the indicated MOI of Ad-GFP and Ad-AC and analyzed for PTEN phosphorylations by western blotting (A). (B) The PTEN C-terminus phosphorylation site mutants A4 (S380A, T382A,T383A,S385A) and E4 (S380E,T382E,T383E,S385E) were transfected into PPC1 along with FLAG-Crm1 and stimulated for 2 hours with 500 nM S1P or PBS. Cell lysates were immunoprecipitated with anti-FLAG beads. (C) The PTEN A4 and E4 were transfected into PPC1, stimulated for 2 hours with 500 nM S1P or PBS, and immunostained for PTEN. Bars represent the percentage of cells with PTEN in the nucleus. Student’s t-test, **p<.01.
(TIF)

Figure S9 **PPC1 cells transfected with WT-PTEN or PTEN-NLS were infected with Ad-GFP or Ad-AC for 48 hours.** A) Cells were immunostained for PTEN, and the percentage of cells which had nuclear PTEN in each treatment is graphed. B) Whole cell lysates were analyzed by immunoblotting. Student’s t-test, **p<.01.
(TIF)
