# Supplementary figures and images for: Acid Ceramidase Promotes Nuclear Export of PTEN through Sphingosine 1-Phosphate Mediated Akt Signaling
Source: PLoS One. 2013 Oct 1;8(10):e76593. doi: 10.1371/journal.pone.0076593 (PMC3788144; doi:10.1371/journal.pone.0076593)

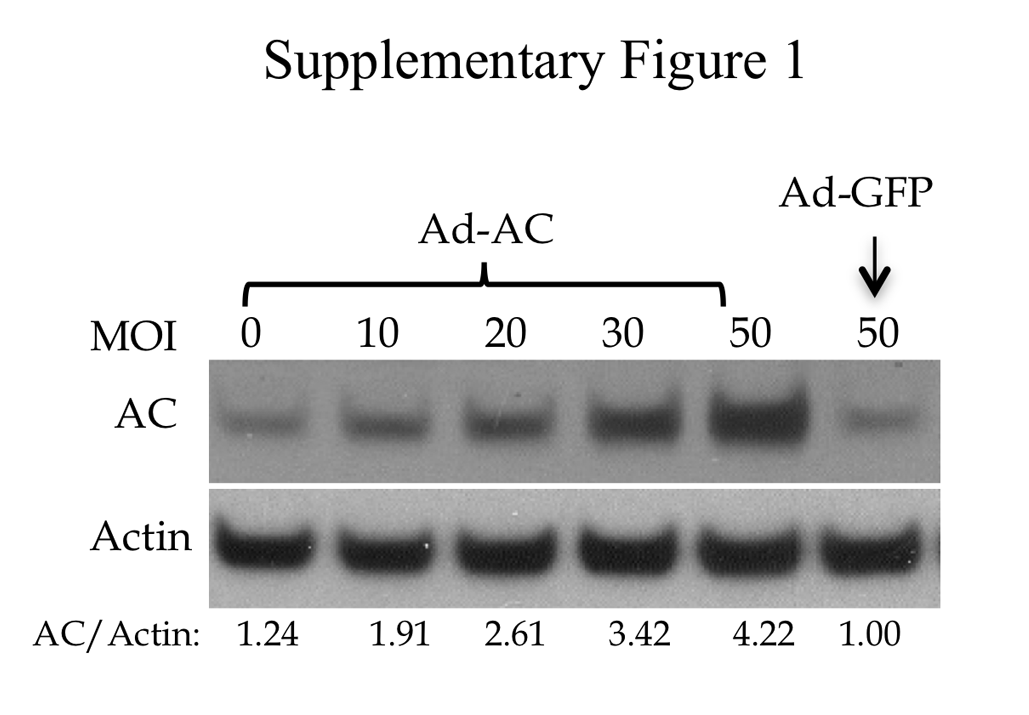

Supplement: Figure S1 — PPC1 cells were infected with Ad-AC at MOI ranging from 0 to 50, or Ad-GFP at MOI 50 and probed for expression of AC. NIH ImageJ was used to measure band densitometries and generate AC/Actin ratios, which were normalized to Ad-GFP MOI 50. (TIF) [file pone.0076593.s001.tif]

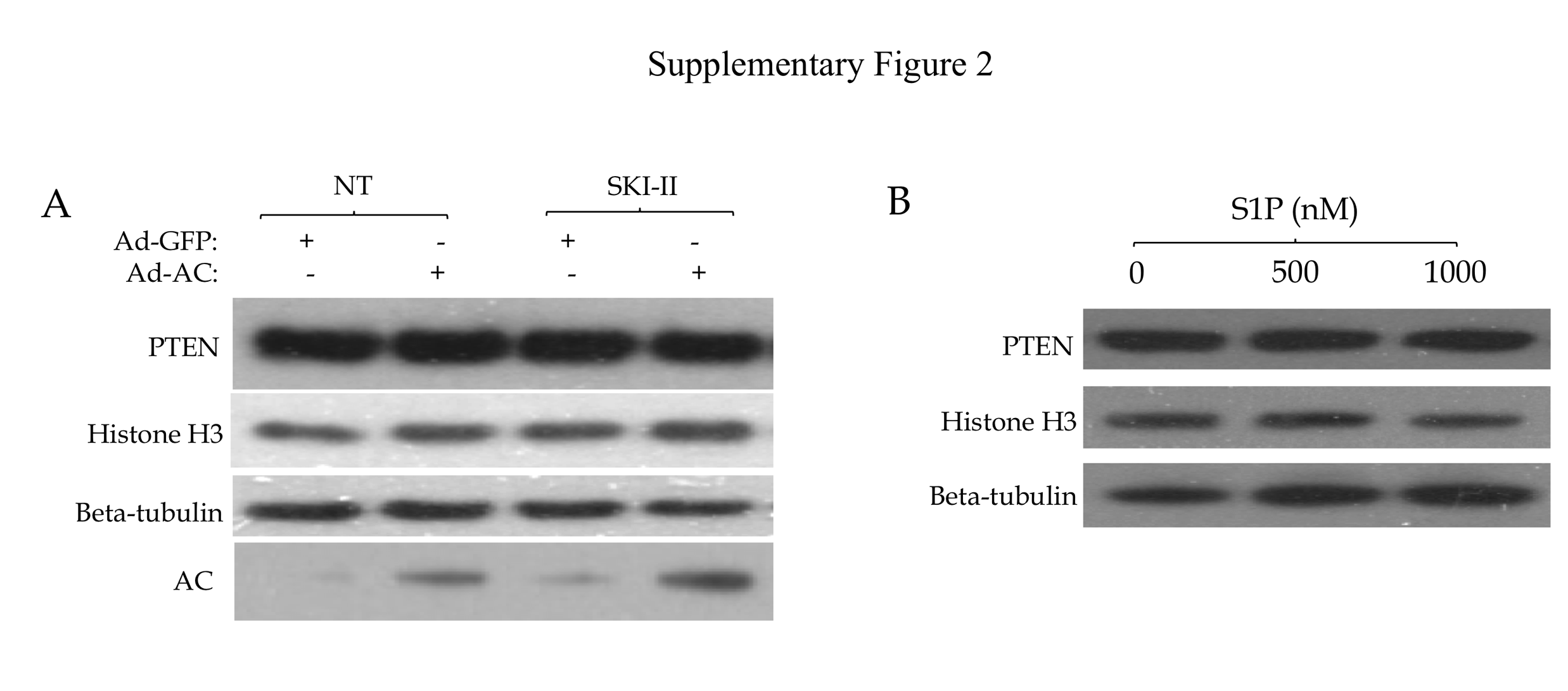

Supplement: Figure S2 — PPC1 cells transfected with WT-PTEN were infected with Ad-GFP or Ad-AC for 48 hours in the presence of DMSO (no treatment; NT) or the sphingosine kinase inhibitor SKI-II for 24 hours (A). Whole cell lysates were analyzed by immunoblotting. (B) PPC1 cells transfected with WT-PTEN were treated with the indicated dose of S1P or PBS for 2 hours. Whole cell lysates were analyzed by immunoblotting. (TIF) [file pone.0076593.s002.tif]

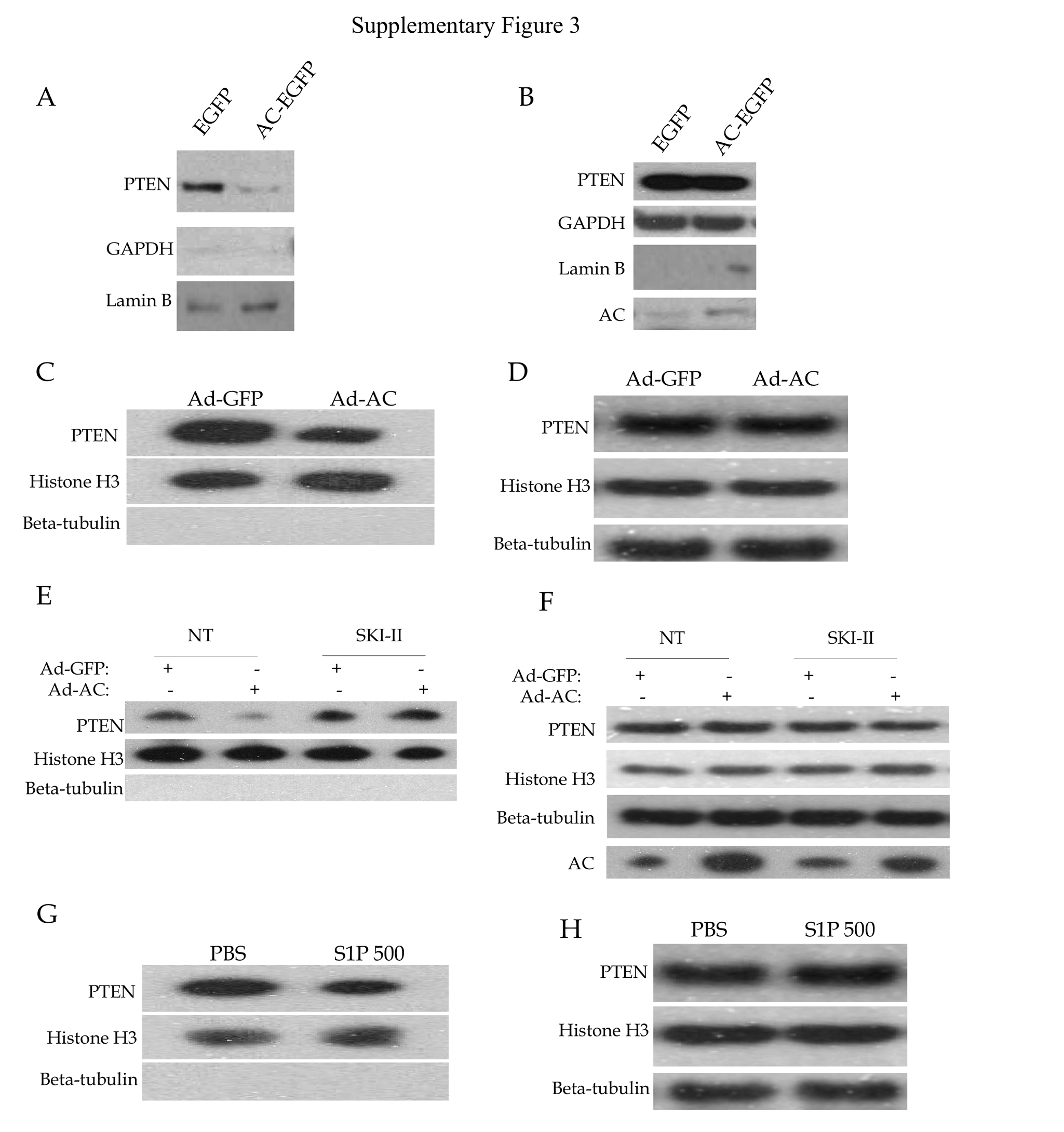

Supplement: Figure S3 — Nuclear fractions (A) and whole cell lysate (B) of DU145 cells stably expressing AC (AC-EGFP and empty vector (EGFP) were collected and analyzed by western blotting. Nuclear fractions (C) and whole cell lysates (D) of DU145 cells infected with Ad-GFP or Ad-AC were collected and analyzed by western blotting. E-F) DU145 cells were infected with Ad-GFP or Ad-AC for 48 hours and treated with SKI-II for 24 hours prior to isolation of nuclear fractions (E) and whole cell lysates (F) and western blot analysis.G-H) DU145 cells were stimulated with 500 nM S1P for 2 hours prior to isolation of nuclear fractions (G) and whole cell lysates (H). (TIF) [file pone.0076593.s003.tif]

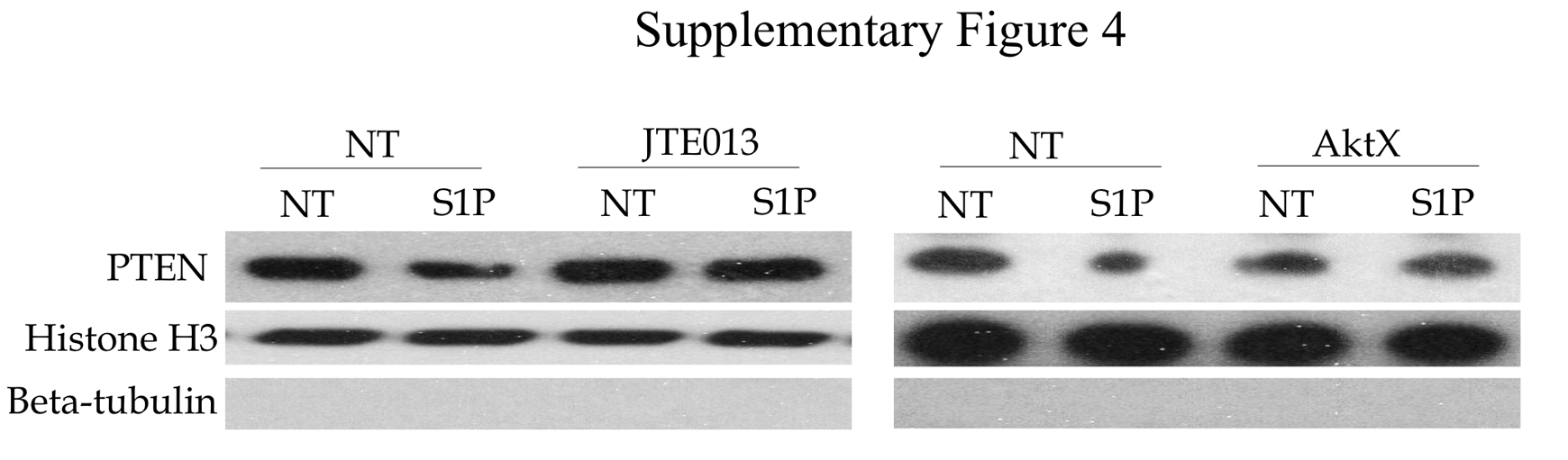

Supplement: Figure S4 — DU145 cells were treated with A) 1µM JTE013 or DMSO (NT) or B) 5µM AktX or water (NT) for 24 hours prior to stimulation with 500 nM S1P or PBS (NT) for 2 hours. Nuclear fractions were analyzed by western blotting. (TIF) [file pone.0076593.s004.tif]

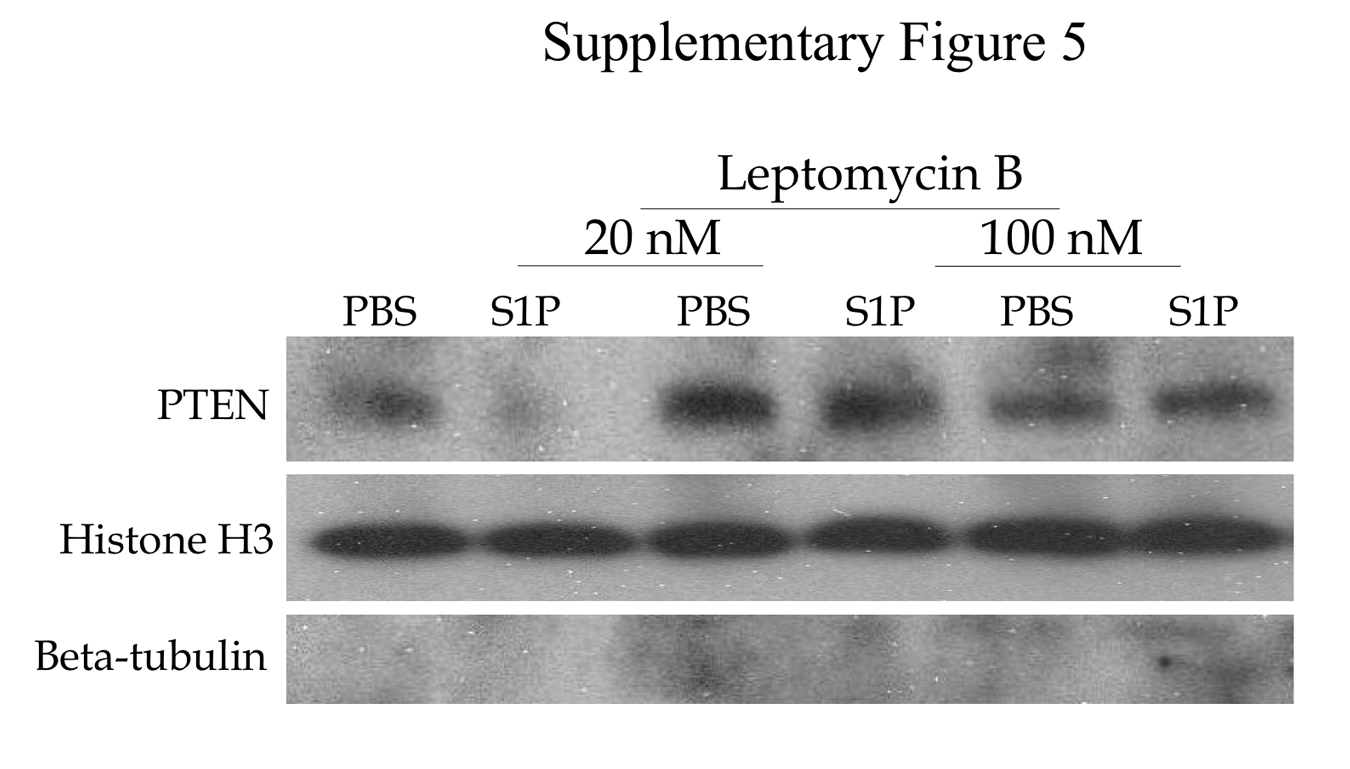

Supplement: Figure S5 — DU145 cells were treated with the indicated concentration of Leptomycin B for 24 hours prior to stimulation with 500 nM S1P for 2 hours. Nuclear fractions were analyzed by western blotting. (TIF) [file pone.0076593.s005.tif]

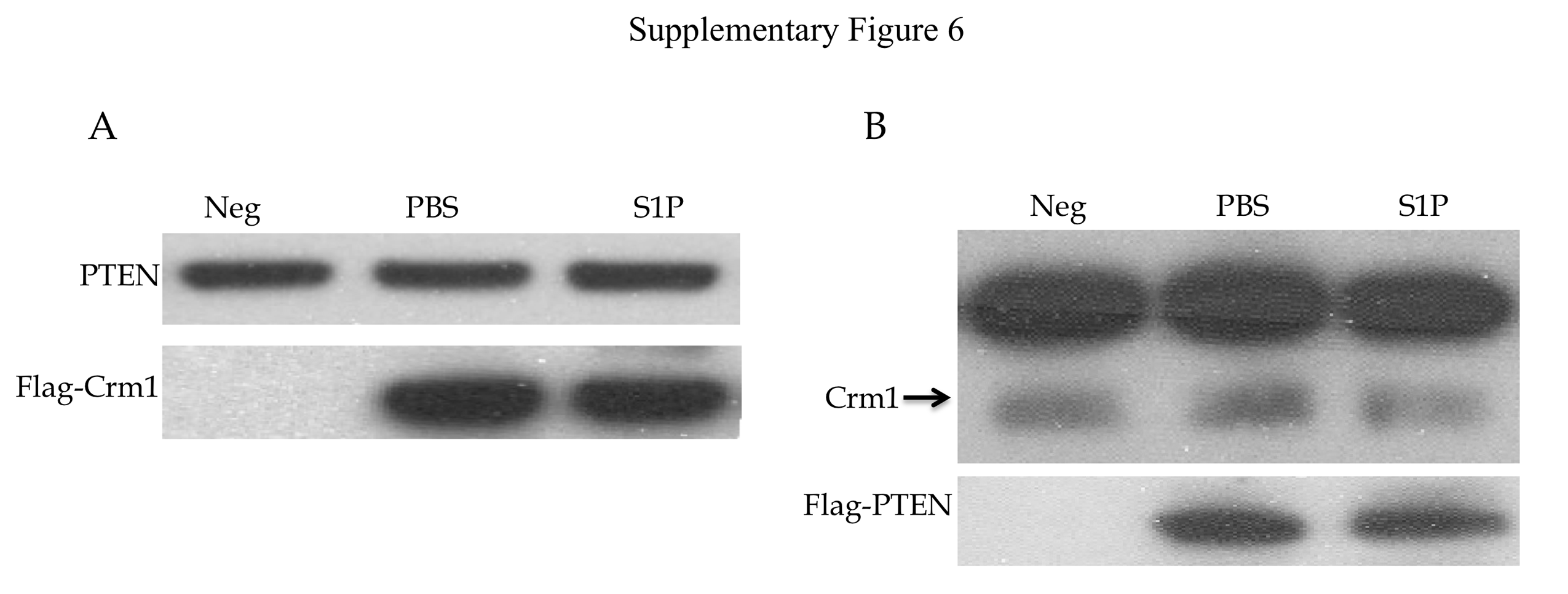

Supplement: Figure S6 — PPC1 cells were transfected with WT-PTEN and FLAG-Crm1 (A). Cells were collected after 2 hours stimulation with 500nM S1P or PBS. The negative control (Neg) indicates lysate from cells not transfected with FLAG-Crm1. (B) PPC1 cells were transfected with FLAG-PTEN and collected after 2 hour stimulation with 500nM S1P or PBS. The negative control (Neg) indicates lysate from cells not transfected with FLAG-PTEN. (TIF) [file pone.0076593.s006.tif]

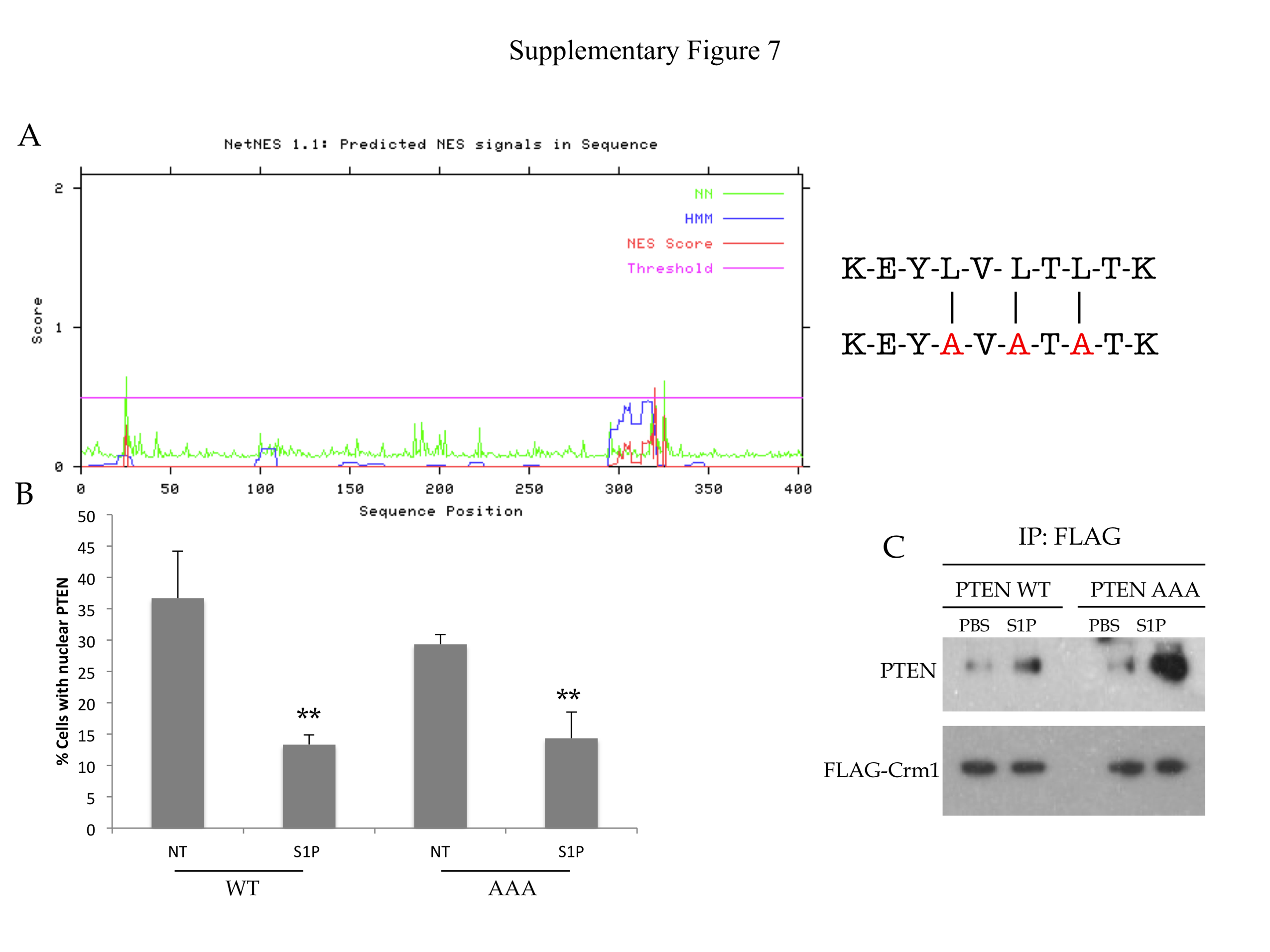

Supplement: Figure S7 — The amino acid sequence of PTEN was analyzed by NetNES1.1 for potential nuclear export signals (A). The identified sequence was mutated (LLL to AAA). (B) WT-PTEN and PTEN-AAA were transfected into PPC1 cells prior to stimulation with 500 nM S1P. Bars indicate the percentage of cells with PTEN in the nucleus. C) PPC1 cells were transfected with FLAG-Crm1 and either WT-PTEN or PTEN-AAA. After 2 hours stimulation with 500 nM S1P, cell lysates were immunoprecipitated with anti- FLAG beads. Student’s t-test, **p<.01. (TIF) [file pone.0076593.s007.tif]

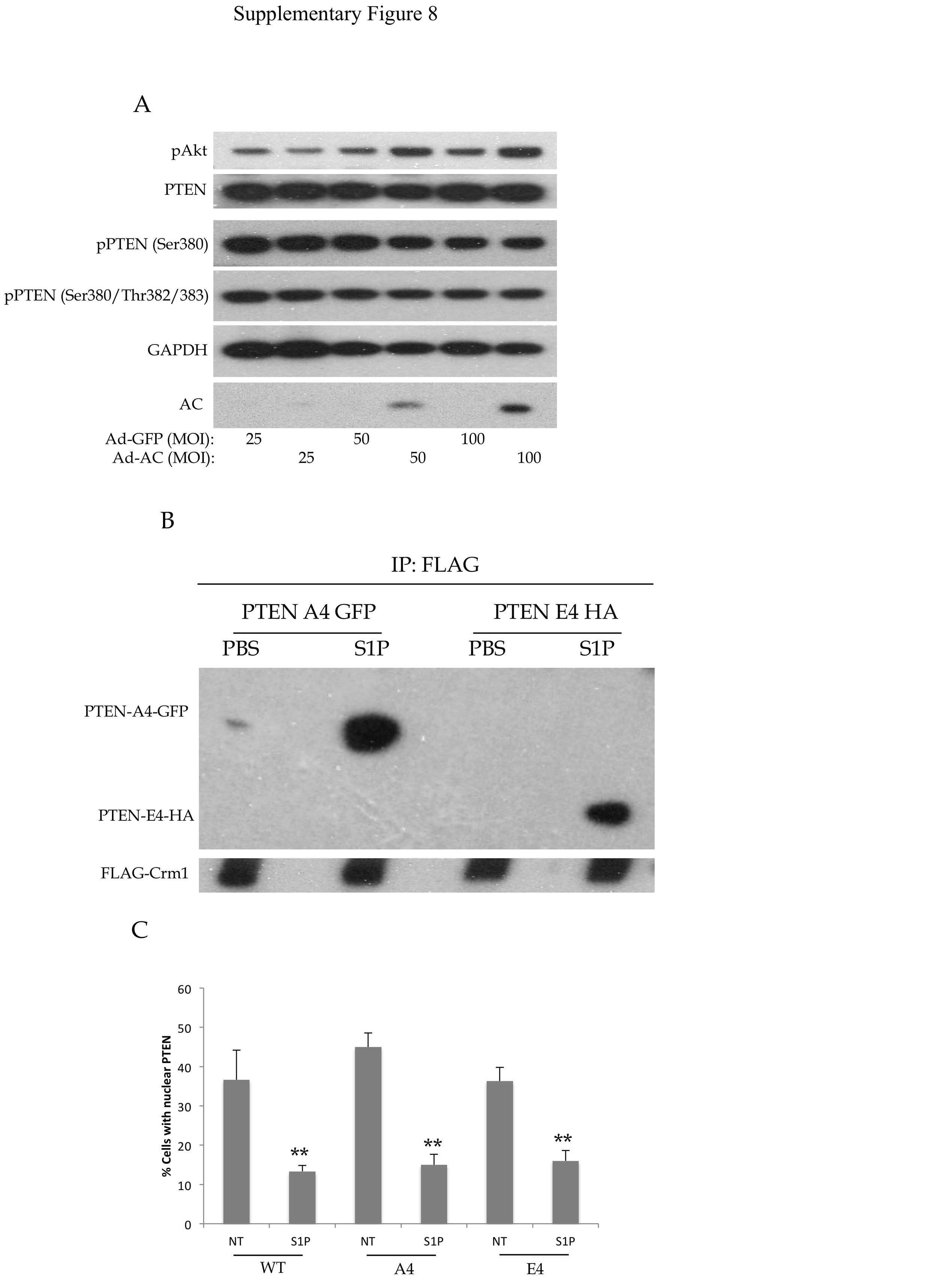

Supplement: Figure S8 — DU145 cells were infected with the indicated MOI of Ad-GFP and Ad-AC and analyzed for PTEN phosphorylations by western blotting (A). (B) The PTEN C-terminus phosphorylation site mutants A4 (S380A, T382A,T383A,S385A) and E4 (S380E,T382E,T383E,S385E) were transfected into PPC1 along with FLAG-Crm1 and stimulated for 2 hours with 500 nM S1P or PBS. Cell lysates were immunoprecipitated with anti-FLAG beads. (C) The PTEN A4 and E4 were transfected into PPC1, stimulated for 2 hours with 500 nM S1P or PBS, and immunostained for PTEN. Bars represent the percentage of cells with PTEN in the nucleus. Student’s t-test, **p<.01. (TIF) [file pone.0076593.s008.tif]

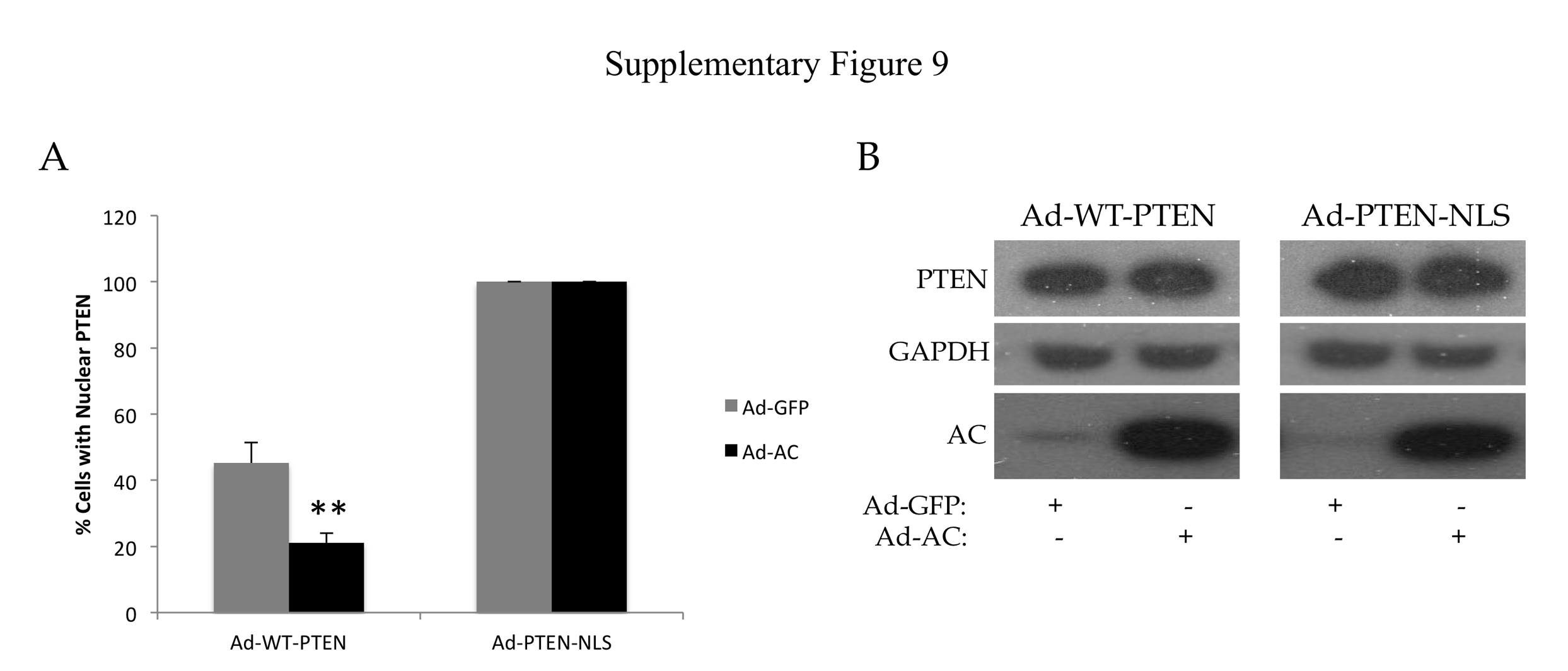

Supplement: Figure S9 — PPC1 cells transfected with WT-PTEN or PTEN-NLS were infected with Ad-GFP or Ad-AC for 48 hours. A) Cells were immunostained for PTEN, and the percentage of cells which had nuclear PTEN in each treatment is graphed. B) Whole cell lysates were analyzed by immunoblotting. Student’s t-test, **p<.01. (TIF) [file pone.0076593.s009.tif]
